# Green Mold of Citrus: Recent Insights into *Penicillium digitatum* Pathogenicity and Biological Control Strategies

**DOI:** 10.1021/acs.jafc.6c02943

**Published:** 2026-06-20

**Authors:** Evandro Silva, Maria Clara Santana Aguiar, Eder de Vilhena Araújo, Paula de França, Roberto G. S. Berlinck, Ana-Rosa Ballester, Luis González-Candelas, Taicia Fill

**Affiliations:** † 28132 State University of Campinas, Institute of Chemistry, Campinas, São Paulo 13083-970, Brazil; ‡ Institute of Chemistry of São Carlos, 153988 University of São Paulo, São Carlos, São Paulo 13566-590, Brazil; § 16379 Instituto de Agroquímica y Tecnología de Alimentos, IATA-CSIC, Paterna, Valencia 46980, Spain; ∥ National Institute of Science and Technology in Human Pathogenic Fungi, São Paulo 04023-062, Brazil

**Keywords:** Penicillium digitatum, biocontrol, fungicide resistance, omics, phytohormones, virulence factors

## Abstract

*P. digitatum*, the causal agent of citrus green mold, remains the most destructive postharvest pathogen of citrus worldwide. Recent advances have greatly expanded our understanding of the molecular dialogue between *P. digitatum* and citrus hosts, revealing coordinated virulence strategies involving cell wall-degrading enzymes, major facilitator superfamily transporters, transcription factors, and secondary metabolism, alongside host defenses mediated by phytohormones and specialized metabolites. This review integrates genomic, transcriptomic, metabolomic, and functional genetic discoveries, including CRISPR/Cas9 and *Agrobacterium tumefaciens*-mediated transformation, which have accelerated the characterization of fungal pathogenicity and host resistance. We further assess biological control as a sustainable alternative to chemical fungicides, emphasizing complementary mechanisms such as niche competition, antibiosis, volatile organic compound (VOC) production, biofilm formation, iron sequestration, lipopeptide synthesis, and induction of host defenses. In addition, we highlight microbiome-informed strategies and the design of synthetic microbial communities (SynComs) as promising next-generation approaches to enhance efficacy, stability, and ecological resilience in citrus postharvest disease management.

## Graphical abstract

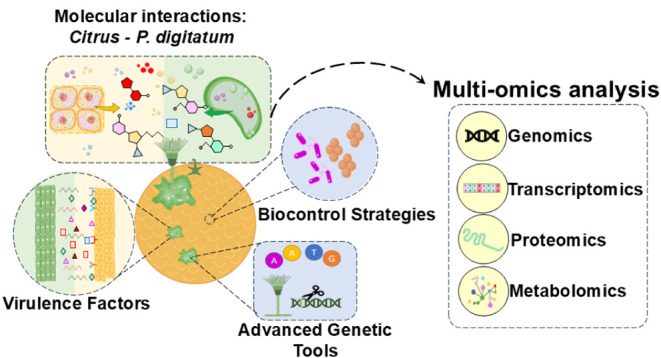

## 1 Introduction

Citrus fruits rank among the most economically valuable and nutritionally important horticultural commodities worldwide, with major producers such as Brazil, China, India, and countries in the Mediterranean basin exporting millions of tons annually.^1^ Within this group, *Citrus sinensis* (L.) Osbeck and other species are widely cultivated across tropical and subtropical regions, playing a central role in local consumption and international trade.^2^ In addition to their economic relevance, citrus fruits are also recognized for their high nutritional value.^3^ They are rich in functional compounds, including dietary fiber, vitamins, polysaccharides, flavonoids, and carotenoids, which contribute to lowering blood lipid levels, providing antioxidant benefits, and reducing the risk of chronic diseases.^4^ However, postharvest fungal diseases pose a major challenge to citrus quality and commercial viability, with losses occurring predominantly during storage and distribution.^5^

Among postharvest pathogens, *Penicillium digitatum*, the causal agent of green mold disease, is the most economically significant pathogen, responsible for up to 90% of storage and transportation losses in citrus.^6^ Its impact is especially severe in arid and subtropical regions, where environmental conditions favor pathogen development. Infection usually occurs through wounds in the fruit peel caused by insect activity, hail, wind, or improper handling, which allow the fungus to bypass natural defense barriers and establish an infection.^7^ Consequently, *P. digitatum* causes rapid fruit deterioration, resulting in substantial economic losses in citrus production and trade.^8^

Infection by *P. digitatum* typically begins at wound sites on the fruit peel, where initial white mycelial growth rapidly develops into characteristic olive-green sporulation as the disease progresses.^8^ Early symptoms include water-soaked depressions in the rind, followed by softening, discoloration, and shriveling of the tissue, accompanied by the development of a characteristic putrid odor in the advanced stages of infection.^9^ Under conditions of high humidity (80–90%) and moderate temperature (23–27 °C), the disease advances rapidly, and complete fruit decay may occur within a few days. Airborne spores are readily dispersed, leading to the contamination of adjacent fruits.^10,11^

Although *P. digitatum* is widely recognized as a phytopathogenic fungus responsible for postharvest diseases in citrus fruits, there are documented reports of pulmonary infections caused by this species in humans, highlighting its potential as an emerging opportunistic pathogen.^12–14^ Although such infections remain rare, their occurrence appears to be associated with predisposing conditions, and the detection of *P. digitatum* in immunocompromised patients is clinically relevant and may pose a significant health risk. This context reinforces the need to monitor and better understand the behavior of *P. digitatum* across different clinical settings, particularly among vulnerable populations.^14^

In addition to direct infection routes, *P. digitatum* may pose a potential public health concern through indirect exposure via citrus fruits and processed products contaminated with fungal secondary metabolites. Araújo et al., demonstrated that tryptoquialanines A and C are produced during infection and can diffuse through orange tissue layers, reaching the endocarp within 5 days postinoculation (dpi).^15^ Moreover, the presence of tryptoquialanines A and B has been reported in commercial orange juices, indicating that these compounds may persist throughout the production chain and reach the final consumer product.^15^ Although the toxicological effects and regulatory limits of tryptoquialanines in food products are not yet fully established, conventional processing treatments such as pasteurization and high-pressure processing have been shown to reduce their levels only partially.^16^ Collectively, these findings highlight the need for further studies to assess exposure levels, toxicological relevance, and potential implications for food safety.

Currently, the management of citrus postharvest diseases relies primarily on chemical fungicides, such as imazalil (IMZ), which belongs to the demethylation inhibitor (DMI) group, and thiabendazole (TBZ).^17^ Although these compounds have long been considered effective, their intensive and recurrent use poses serious concerns. Beyond the potential risks to human health and environmental safety, the selective pressure exerted by these chemicals has accelerated the emergence of fungicide-resistant strains of *P. digitatum*.^18^ This scenario underscores the urgent need for sustainable and innovative control strategies, including biological control agents and integrated management approaches, to reduce dependence on synthetic fungicides and ensure long-term effectiveness in citrus disease management.^19^

The rising concern about the detrimental effects of *P. digitatum* on citrus production has stimulated comprehensive studies focused on the *P. digitatum*–citrus interaction. Growing interdisciplinary research efforts have advanced our understanding of *P. digitatum* biology and driven the search for sustainable control strategies. Recent studies have employed diverse approaches, from the characterization of chemical interactions between the fungus and citrus tissues to the development of biocontrol methods using beneficial microorganisms.^20^ In parallel, complete genome sequencing of different *P. digitatum* strains has enabled a deeper exploration of the virulence factors and molecular mechanisms underlying infection, opening new perspectives for targeted management strategies in citrus production.^21–24^

Over the last 5 years, remarkable progress has been made in understanding the *P. digitatum*–citrus interaction, driven by the rapid expansion of omics technologies and advanced molecular tools. These recent findings build upon decades of foundational research on fungal physiology, pathogenicity, and fruit defense responses, revealing new layers of complexity that were previously inaccessible. This review focuses on studies published within the last 5 years, while also acknowledging foundational research on fungal physiology and genomics. It synthesizes these cutting-edge discoveries and emphasizes how they refine, expand, or challenge established knowledge of *P. digitatum* virulence mechanisms, host responses, and sustainable control strategies.

Accordingly, this review integrates the most representative advances of this period, gathering recent evidence on the pathogen’s biology, its virulence determinants, host defense responses, and the development of safer and environmentally sustainable control alternatives. Together, these findings provide a comprehensive and updated view of the dynamic fruit–pathogen–environment interaction, reinforcing the importance of integrative approaches to mitigate green mold losses and promote a more resilient and sustainable citrus industry in the future.

## 2 The *Penicillium digitatum*-Citrus Pathosystem

The interaction between *P. digitatum* and its citrus host is a dynamic process shaped by a complex interplay of chemical signals, physiological responses, and environmental factors. These factors collectively modulate disease progression by either promoting fungal colonization or triggering the fruit’s defense mechanisms.

This section analyzes recent advances in understanding the molecular dialogue within this pathosystem. It addresses the direct mechanisms that influence infection, as well as the indirect factors that alter the host’s physiological response or the surrounding environment. Understanding these communication pathways is essential for developing effective and sustainable control strategies for green mold disease, which is a significant postharvest challenge in the citrus industry.

### 2.1 Host Defense Mechanisms

Extensive research on plant–pathogen interactions has revealed key mechanisms underlying fungal infections, enabling the development of effective and environmentally sustainable control strategies. Plants have evolved robust immune systems that encompass both innate (constitutive resistance) and acquired (inducible resistance) responses to resist pathogen invasion and cope with diverse biotic stresses.^25,26^

In citrus fruits, defense is primarily initiated by a complex repertoire of constitutive biochemical barriers concentrated in the peel, comprising volatile organic compounds and phenolic metabolites with widely reported antimicrobial properties (Figure 1).^27^ Citrus essential oils, mainly composed of terpenes such as limonene, α-pinene, and γ-terpinene, are known to disrupt fungal cell membranes and inhibit pathogen growth.^28^ In parallel, carotenoids, phenols, flavonoids, and coumarins act as potent antifungal compounds by interfering with cellular processes and enhancing antioxidant capacity.^28,29^

**Figure 1:**
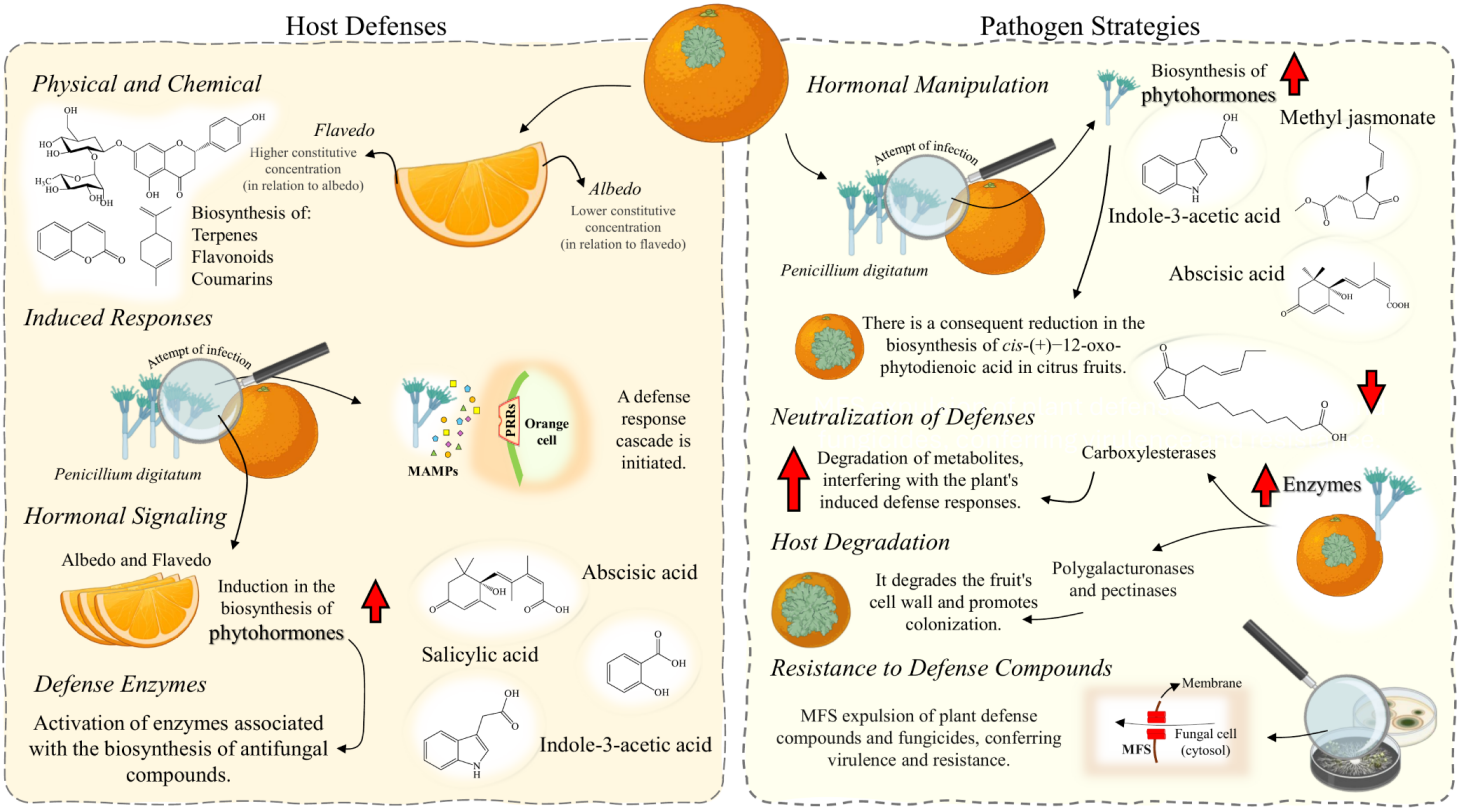
Overview of the molecular mechanisms involved in the *Penicillium digitatum*–citrus interaction. Citrus fruits exhibit both constitutive and inducible defense responses. Pattern recognition receptors (PRRs) detect microbe-associated molecular patterns (MAMPs), triggering downstream defense signaling, such as oxidative burst, hormone regulation, and expression of pathogenesis-related (PR) proteins. These responses involve the accumulation of reactive oxygen species (ROS), phenylpropanoids, and secondary metabolites that reinforce cell walls and restrict pathogen invasion. On the fungal side, *P. digitatum* employs virulence factors, including cell wall-degrading enzymes, transcription factors, and major facilitator superfamily (MFS) transporters that export antifungal compounds and mitigate oxidative stress.

The efficacy of these antifungal substances is associated with metabolic reprogramming in citrus tissues during pathogen interaction rather than the activation of a single specific pathway. For instance, Zeng et al. demonstrated that the treatment of *Citrus reticulata* fruits with the flavonoid naringin delayed fruit deterioration by modulating oxidative stress, particularly through the reduction of hydrogen peroxide (H_2_O_2_) levels and the enhancement of antioxidant-related responses.^29^ These findings suggest that flavonoids contribute to fruit resistance by regulating redox homeostasis rather than directly activating a defined biosynthetic pathway.

Similarly, Fernandes et al. showed that resistance in *Citrus latifolia* is associated with the accumulation of phenolic compounds, especially coumarins, following pathogen challenge.^30^ Importantly, individual coumarins did not exhibit strong antifungal activity, whereas a coumarin-rich fraction displayed enhanced inhibitory effects, indicating that their role in defense relies on synergistic interactions among multiple metabolites.^30^ This highlights that citrus defense mechanisms are based on complex metabolic networks rather than the actions of single compounds or pathways.

Within this framework, the coordination of these defense-related metabolites is closely linked to primary metabolism, which provides the energy and metabolic precursors required to sustain these responses. In particular, central carbon metabolism, including the tricarboxylic acid (TCA) cycle, plays a key role in supporting defense by supplying carbon skeletons and energy for the biosynthesis of secondary metabolites.^31^ In addition to sustaining biosynthetic demands, metabolic reprogramming during pathogen infection has been associated with changes in volatile compound production, including esters, which may contribute to plant defense and signaling processes.^32^ Infection by *P. digitatum* has also been reported to alter ester profiles in citrus tissues, suggesting a link between pathogen-induced metabolic shifts and the emission of defense-related volatiles. Together, these interconnected metabolic pathways support the activation of transcription factors, signaling molecules, and pathogen-related proteins that collectively contribute to the establishment of plant resistance.^28^

This enhanced defense capacity is particularly evident in the flavedo, the outer layer of the fruit, which is significantly more resistant to pathogen invasion than the inner albedo. This increased resistance is associated with a more robust constitutive and inducible defense system, characterized by elevated secondary metabolism and the rapid activation of reactive oxygen species (ROS)-related responses.^28^

Intense secondary metabolism in the flavedo leads to the accumulation of potent antimicrobial metabolites, such as phenolic compounds and flavonoids, which act as preformed chemical barriers against fungal growth.^33^ Furthermore, the defense system is actively regulated by key transcription factors, such as CsMYB96, which enhance the defense state by activating salicylic acid (SA) biosynthesis and promoting the production of defense-related phenolic metabolites.^34^ This highlights the central role of the SA pathway in fruit defense, acting as a primary hormonal signal during infection. SA-mediated signaling is also critical for the hypersensitive response, enabling the fruit to restrict pathogen spread through localized programmed cell death.^35^ Together, these interconnected pathways form a sophisticated, multilayered defense system that remains constantly active and provides foundational protection even in the absence of a direct pathogen challenge.

### 2.2 Activation and Overcoming of Inducible Defenses

When a pathogen breaches or bypasses a plant’s constitutive barriers, inducible defense mechanisms are swiftly activated. This process typically starts with the identification of conserved microbe-associated molecular patterns (MAMPs) by pattern recognition receptors (PRRs), which reside on the surface of plant cells (Figure 1). In the case of *P. digitatum*, citrus PRRs recognize fungal cell wall components, such as chitin, triggering MAMP-triggered immunity (PTI). PTI then initiates a cascade of defense responses involving the activation of complex signaling pathways, rapid ion fluxes, and transcriptional reprogramming, leading to the expression of numerous defense-related genes.^36,37^ These responses aim to limit pathogen growth and spread.

However, through coevolution with their host plants, many pathogens, including *P. digitatum*, have developed sophisticated adaptive advantages that allow them to suppress or evade host immunity.^38^ *P. digitatum* has a remarkable ability to manipulate the host environment to its benefit. It not only avoids inducible defenses but also actively suppresses them to create a favorable niche for proliferation. For example, to overcome the production of esters from the previously mentioned citrus wounds, *P. digitatum* secretes enzymes such as carboxylesterase that break down esters into organic acids. The accumulation of these acids rapidly lowers the pH of the wound, creating optimal conditions for the effective functioning of the cell-wall-degrading enzymes (CWDEs) of the fungus.^32,39^

These CWDEs, including polygalacturonases and pectinases, are essential for depolymerizing the host’s cell wall components. This process leads to tissue maceration and facilitates nutrient uptake.^40,41^ Recent transcriptomic evidence shows that the expression of these enzymes is tightly regulated and significantly increased in highly virulent strains during the early stages of citrus infection. This underscores their role as primary drivers of fungal colonization.^42^ This facilitates pathogen colonization and proliferation in the host.

A critical PTI response in citrus fruits is the oxidative burst, which is characterized by the rapid production of ROS, such as H_2_O_2_. ROS act as direct antimicrobial agents that damage pathogen structures and as crucial signaling molecules that propagate defense signals to other parts of the plant. This leads to the induction of pathogen-related (PR) genes and the synthesis and accumulation of lignin and/or suberin, strengthening the cell wall.^43,44^

Studies have shown that during *P. digitatum* infection, H_2_O_2_ production is significantly lower than that during interactions with other pathogens. This suggests that the fungus actively suppresses this vital defense mechanism.^45^ This suppression compromises one of the plant’s most important defense mechanisms, allowing the fungus to evade oxidative damage and continue to colonize. These findings imply that *P. digitatum* has the ability to either neutralize host ROS or inhibit their production. Lin et al., demonstrated that the fungal virulence effector, PdCDIE1 (*Penicillium digitatum* Cell Death-Inducing Effector 1), is secreted by the pathogen *P. digitatum* to disrupt ROS homeostasis and host defense.^46^ PdCDIE1 accomplishes this by interfering with the plant’s Ca^2+^ signaling and disrupting the connection between calmodulin (CaM), the primary Ca^2+^ sensor, and the citrus Hsp70 protein. This deregulates the defense equilibrium, resulting in cell death.^46^

Recent studies on microbial defense mechanisms have also shown that *PdMFSs* genes play an important regulatory role, as reported by de Ramón-Carbonell and Sánchez-Torres.^47^ These genes encode transporters belonging to the Major Facilitator Superfamily (MFS), which are implicated in the infectious capacity of *P. digitatum* by mediating the efflux of host-derived protective compounds. In this way, *P. digitatum* uses MFS transporters to expel toxic molecules that accumulate within its cells. This increases its tolerance to the oxidative stress compounds generated by the host.^47^ These findings demonstrate that these transporters confer resistance to multiple fungicides and play a direct role in fungal virulence. This mechanism is essential for the fungus’s survival within the host. This is a significant discovery because it links fungicide resistance to a fundamental virulence mechanism, providing two potential targets for disease control.

### 2.3 Interfering with Wound-Induced Defenses and Hormone Signaling

Another plant defense mechanism that *P. digitatum* has evolved to circumvent is the wound-induced release of esters, such as 3-hexenyl acetate, which signals the initiation of the jasmonic acid (JA) defense pathway. As mentioned earlier regarding pathogen colonization (Section 2.2), *P. digitatum* secretes carboxylesterases that break down increased citrus wound esters into carboxylic acids.^32,39^ This mechanism provides the pathogen with two advantages: it rapidly reduces the pH of the wound, which optimizes the activity of fungal cell wall-degrading enzymes, and it directly interferes with the plant’s JA signaling cascade. By removing the precursor signal (esters), the fungus prevents the proper accumulation and activation of the JA-mediated immune response, thereby bypassing a key line of defense against the infection.^32^

Furthermore, *P. digitatum* employs a strategy to obtain essential nutrients for its metabolism by hydrolyzing the citrus wound proteins. It does so by secreting ubiquitin, ubiquitin-coupled enzymes, proteasomes, and other lysis enzymes. This process predominantly occurs within the first 48 h after *P. digitatum* comes into contact with citrus wounds. This period is recognized as critical for the fungus to successfully establish an infection.^32^ This rapid enzymatic degradation provides the fungus with a ready supply of amino acids while simultaneously breaking down host-defense proteins.^34^

As mentioned before, the active acidification process triggered by *P. digitatum* optimizes the activity of polygalacturonases and pectinases involved in host cell wall degradation. These enzymes soften the peel and break down the structural integrity of citrus fruits, facilitating fungal colonization. Li et al., demonstrated that this process is regulated by the plasma membrane H ± ATPase (PMA1).^10^ PMA1 plays a significant role in *P. digitatum* cell growth and pathogenicity by regulating the biosynthesis of cell wall components and membrane ergosterol, thereby promoting the activity of these lytic enzymes. This highlights the fungus’s ability to actively modify the immediate environment to enhance its virulence.^10^

### 2.4 Fungal Manipulation of the Plant’s Phytohormones and Modulation of the Host’s Defense Signaling Network

The coevolution of *P. digitatum* and citrus plants involves a complex interplay between the pathogen and the plants’ phytohormones. The fungus directly influences the production and levels of key hormones, such as salicylic acid (SA), abscisic acid (ABA), and indole-3-acetic acid (IAA), within the fruit (Figure 1). These changes can profoundly affect the plant’s response to infection.^48^

For example, ABA protects citrus fruits against *P. digitatum* infection. Comparative analyses between “Navelate” oranges, which are rich in ABA and more resistant, and the mutant “Pinalate,” which has low levels of ABA and is less resistant, demonstrated this protective role.^48^ The researchers demonstrated that ABA deficiency in the Pinalate mutant is associated with repressed constitutive defense mechanisms, including the metabolism of terpenoids, phenylpropanoids, and glutathione. Additionally, oxidation–reduction processes and signaling pathways related to plant hormone defense were repressed. Conversely, applying exogenous ABA to the “Pinalate” mutant reduced its susceptibility to infection by decreasing the area of lesions, mycelium, and sporulation development to levels similar to those of the “Navelate” variety.^48^

In a complementary study, the protective role of abscisic acid (ABA) in citrus fruits has been associated with the modulation of phospholipase D (PLD) and phospholipase C (PLC) activities, which are two key enzymes in plant signaling pathways. The activation of these phospholipases represents a critical step in the defense cascade, and the capacity of citrus fruits to trigger this response is closely related to the presence of ABA.^28^

A recent study has shown that the interaction between phytohormones and the defense responses of citrus fruits against*P. digitatum* is both tissue-specific and highly complex.^49^ Notably, indole-3-acetic acid (IAA) plays a role in the early recognition of infection, as evidenced by a significant yet transient increase in IAA levels in the flavedo of “Navelate” oranges at the onset of infection. This response, however, was absent in the albedo and in the flavedo of the ABA-deficient mutant “Pinalate”. These findings indicate that IAA signaling contributes specifically to the initial pathogen detection in the flavedo and that its regulation is closely intertwined with the presence of ABA.^49^

Additionally, the study demonstrated that SA plays a fundamental role in the initial defense signaling of both the flavedo and the albedo. The discovery that the fungus produces various phytohormones, including ABA, SA, and IAA, suggests that *P. digitatum* can manipulate the host’s hormonal environment to its advantage. The interconnection between the SA and ABA signaling pathways was revealed, indicating that citrus resistance relies on a network of intricate hormone interactions rather than a single hormone.^49^ These interactions manifest differently in the various layers of the fruit. Furthermore, applying SA to citrus fruits significantly increases the activity of essential defense enzymes, including phenylalanine ammonia-lyase (PAL), peroxidase (POD), and polyphenol oxidase (PPO). PAL is particularly important because it is a key enzyme in the phenylpropanoid pathway. It synthesizes phenolic compounds that act as antimicrobial barriers.^50^

In addition to its interactions with IAA, SA, and ABA, the study also provided new information about jasmonates, such as cis-(+)-12-oxo-phytodienoic acid (OPDA), methyl jasmonate (MeJA), and jasmonoyl-isoleucine (JA-Ile). During the initial phase of infection, a significant decrease in OPDA levels was observed. OPDA is a precursor in the jasmonate synthesis pathway. This decline may be a mechanism by which the pathogen suppresses the plant’s initial defense responses. In later stages, however, the fungus appears to actively modulate jasmonate metabolism, redirecting it to produce MeJA and JA-Ile.^49^

The fungus’s ability to produce these active hormonal compounds is a groundbreaking discovery in the field of study and suggests a pathogen’s sophisticated strategy for manipulating the host. The fungus produces IAA and jasmonates when interacting with the host tissue, indicating a bidirectional molecular dialogue in which the fungus senses and actively modifies the plant’s physiological environment. These findings further support the idea that the relationship between *P. digitatum* and citrus involves a sophisticated molecular response in which both organisms use phytohormone signaling modulation as a critical strategy to influence the infection outcomes.

### 2.5 Secondary Metabolites Produced by *Penicillium digitatum* During Pathogen-Host Interaction

During citrus infection, *P. digitatum* synthesizes a wide array of secondary metabolites, whose production is tightly regulated by environmental cues and direct contact with host tissues. These compounds, including indole alkaloids, terpenes, peptides, and aromatic analogs, play pivotal roles in the fungus’s physiological adaptation and ecological competitiveness in postharvest environments.^51^ Many of these metabolites also exhibit antimicrobial and cytotoxic activities, stimulating growing interest in their chemical and functional characterization.^52^

Using mass spectrometry imaging (MSI) combined with GNPS molecular networking, Costa et al. mapped in situ the distribution of secondary metabolites directly on infected orange peels between 4- and 7-dpi.^53^ The study revealed localized accumulation of compounds associated with the tryptoquialanine biosynthetic pathway, including tryptoquialanine A (1), tryptoquialanine B (2), and their intermediates, tryptoquialanone (3), 15-dimethyl-2-epi-fumiquinazoline A (4), and deoxytryptoquialanone (5). Additionally, tryptoquivaline L (6), tryptoquivaline Q (7), fumiquinazoline A (8), and fumiquinazoline C (9) were identified in *P. digitatum* for the first time, along with tryptoquialanine C (10), a novel indole alkaloid structurally related to tryptoquialanine A. Bioassays revealed a strong insecticidal activity of tryptoquialanine A against the mosquito *Aedes aegypti*, suggesting that this molecule plays a broader ecological role associated with chemical defense and environmental competition rather than acting as a direct virulence factor.^53^ This interpretation is supported by genetic evidence showing that the *tqaA* gene, which is essential for the biosynthesis of tryptoquialanine A, is not required for citrus fruit infections.^51^

In this context, *P. digitatum* likely produces tryptoquialanines as part of an ecological adaptation strategy, contributing to microbial competition in the postharvest environment and defense against natural competitors and predators, such as insects or mites, which could degrade the mycelium or compete for the same substrates. Thus, rather than functioning as a virulence determinant, these alkaloids may act as trophic interaction metabolites, enhancing the fungus’s ability to survive and persist in organic-rich niches, such as citrus fruit surfaces. Collectively, these findings highlight MSI-guided molecular networking as a powerful approach to connect spatial metabolite distribution with biosynthetic pathways and potential ecological and biological functions of *P. digitatum* metabolites during citrus fruit decay.^53^

Building upon this, Costa et al., demonstrated that *P. digitatum* actively exports these alkaloids via extracellular vesicles (EVs) during infection (Figure 2).^54^ LC–HRMS and GNPS analyses confirmed that EVs carry tryptoquialanines (1) and (2) and several biosynthetic intermediates, along with fungisporin-type tetrapeptides, namely cyclo-(Phe–Phe–Val–Val) (fungisporin) (11), cyclo-(Phe–Val–Val–Tyr) (12), and their linear analogs Phe–Val–Val–Phe (13) and Phe–Val–Val–Tyr (14).^54^ Bioassays demonstrated marked phytotoxic effects, with tryptoquialanine A completely inhibiting *Citrus sinensis* seed germination and EVs inducing necrotic lesions similar to infection symptoms. Together, these findings indicate that *P. digitatum* employs a sophisticated vesicle-mediated secretion system to deliver indole alkaloids and cyclic peptides that may contribute to virulence and host tissue damage, although their precise role as direct virulence determinants remains to be fully elucidated.^54^

**Figure 2:**
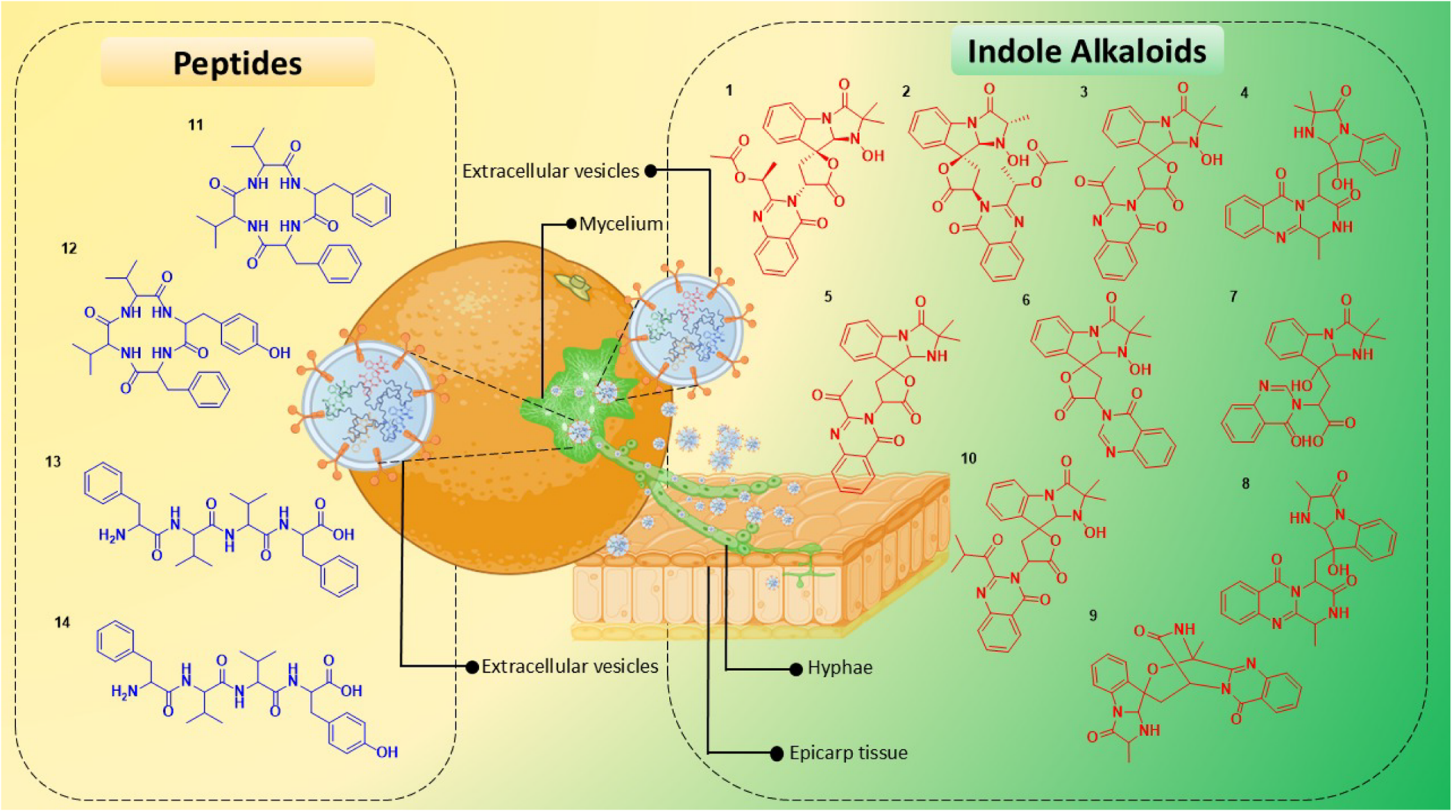
Vesicle-mediated delivery of secondary metabolites during *P. digitatum* pathogenesis in citrus fruit. The illustration shows an infected citrus fruit with a magnified cross-section of the rind, revealing fungal hyphae colonizing host tissue. Blue spherical structures represent extracellular vesicles released by fungal hyphae, mediating the targeted delivery of bioactive secondary metabolites to host cells at the infection site. Fungal secondary metabolites are color-coded according to their chemical class: red structures represent indole alkaloids, including tryptoquialanine A (1), tryptoquialanine B (2), tryptoquialanone (3), 15-dimethyl-2-epi-fumiquinazoline A (4), deoxytryptoquialanone (5), tryptoquivaline L (6), tryptoquivaline Q (7), fumiquinazoline A (8), fumiquinazoline C (9); blue structures represent tetrapeptides, including cyclic forms cyclo-(Phe-Phe-Val-Val) (11) and cyclo-(Phe-Val-Val-Tyr) (12), and linear analogs Phe-Val-Val-Phe (13) and Phe-Val-Val-Tyr (14).

Although deletion of the *tqaA* gene, which is responsible for tryptoquialanine biosynthesis, does not abolish fungal virulence,^51^ the bioactivity profiles of these metabolites suggest a predominant ecological role, acting as chemical weapons that enhance *P. digitatum* competitiveness and adaptation within its environmental niche.^53,54^ In this context, the distinction between metabolites involved in ecological competition, toxicity, and pathogenicity becomes increasingly blurred, highlighting the need for integrative studies that combine metabolomics, genomics, and functional assays to unravel the multifaceted roles of *P. digitatum* secondary metabolites in both host infection and food safety.^20^

Complementary to these in vivo findings, Araújo et al. developed a validated LC–MS/MS method for the quantitative detection of tryptoquialanine A and B in orange juice samples.^16^ This analytical approach confirmed the persistence of *P. digitatum*-derived metabolites in processed citrus products, underscoring the importance of monitoring fungal secondary metabolites as potential chemical markers of contamination and mycotoxin-related safety risks.^16^

More recently, beyond indole alkaloids, a genetic and functional connection between the fumiquinazoline (FQ) biosynthetic pathway and *P. digitatum* virulence was demonstrated. Liu et al., identified two FQ biosynthetic gene clusters distributed across different chromosomes and generated knockout mutants for seven candidate genes, including NRPSs and FAD-dependent flavoproteins.^55^ LC–MS analyses revealed that these fumiquinazoline clusters primarily regulate the biosynthesis of tryptoquialanine A, as evidenced by the disappearance or strong reduction of tryptoquialanine A peaks in six mutants and the accumulation of intermediates such as fumiquinazoline F and deoxytryptoquialanone, indicating a metabolic crosstalk between the fumiquinazoline and tryptoquialanine A pathways. Functionally, the mutants exhibited delayed onset of green mold and, in some cases, significantly smaller lesion diameters. The authors also observed increased sensitivity to UV–C radiation and altered tolerance to low-temperature and KCl stress, implying that these metabolites contribute to the environmental fitness of the pathogen in postharvest settings. Collectively, this study positions the FQ clusters as regulatory hubs integrating secondary metabolism (notably tryptoquialanine A biosynthesis), virulence, and stress responses, offering new targets for green control strategies and chemical markers of contamination.^55^

Overall, the complex molecular dialogue between *P. digitatum* and citrus fruit highlights a highly coordinated interplay between host defense mechanisms and pathogen virulence strategies. The ability of the fungus to manipulate host metabolism, suppress inducible defenses, modulate phytohormone signaling, and produce bioactive secondary metabolites underscores the multifactorial nature of citrus infection. From an applied perspective, these insights provide a valuable framework for the development of innovative and more effective postharvest disease management strategies, including targeted interference with fungal virulence factors, enhancement of host resistance pathways, and the exploration of metabolite-based or microbiome-driven approaches. Ultimately, advancing the understanding of these host–pathogen interactions is essential to reduce postharvest losses, improve fruit quality, and promote more sustainable practices within the citrus industry.

## 3 Genomic Approaches and Functional Gene Analysis in *Penicillium digitatum*-Citrus Fruit Interaction

Genomic approaches and functional gene analysis are important tools for understanding the molecular mechanisms of virulence and the growing resistance to postharvest fungicides in the fungus *P. digitatum*.^17^ The first high-quality genome sequencing in 2012^22^ significantly propelled this genomic era for the pathogen, providing an essential blueprint for subsequent molecular studies. In recent years, significant advances have been achieved in the generation of even more refined genomic assemblies and curated databases, along with the development of molecular tools that enhance functional genomic studies in this species.

Recent studies have generated near-complete genome assemblies of *P. digitatum* using hybrid sequencing approaches that combine short Illumina reads with long-read technologies, such as PacBio or Oxford Nanopore, and chromosome-level scaffolding through Hi-C.^24^ These high-quality assemblies have improved the resolution of repetitive regions and biosynthetic gene clusters (BGCs), offering deeper insights into genes involved in secondary metabolism, virulence, and fungicide resistance.^24,56^

Functional genomics has also advanced substantially with the implementation of targeted gene-editing systems. The CRISPR/Cas9 platform, successfully adapted to *P. digitatum*, has achieved editing efficiencies of up to 80% by optimizing selection strategies and delivery systems.^57^ CRISPR-based knockouts and marker-free systems now complement conventional transformation techniques, such as *Agrobacterium tumefaciens*-mediated transformation (ATMT), which remains a robust and widely used approach for stable gene insertion and random T-DNA insertion mutagenesis.^57,58^ Together, these tools enable the precise functional validation of genes potentially involved in virulence, drug resistance, and adaptation to host environments.

Multiomic analyses, including transcriptomics, metabolomics, and comparative genomics, have expanded the understanding of *P. digitatum* biology beyond single-gene studies. RNA-seq profiling during citrus infection has identified gene networks activated in early infection stages, including those encoding cell-wall-degrading enzymes,^23^ detoxification systems,^59^ and membrane transporters.^47^ A comparison of *P. digitatum* and *P. expansum* genome sequences showed a lower genetic variability in *P. digitatum*,^60^ compatible with a recent clonal expansion of a single lineage of this pathogen. Comparative genomic analyses across *Penicillium* species have also highlighted the diversification of gene families related to host specificity and secondary metabolism, shedding light on how *P. digitatum* evolved as a specialized citrus pathogen.^56^

In the comparative genomic study conducted by Petersen et al., 93 isolates were newly sequenced using long-read technology and integrated with 11 reference genomes, including *P. digitatum* (strains DSM62840 and PdW03).^56^ The resulting pangenome indicated that half of the average number of gene content is shared, while the remaining half represents a variable and adaptive repertoire. Functionally, the accessory genome was significantly enriched in genes associated with secondary metabolite biosynthesis, transmembrane transport, and heme binding, whereas the core genome was dominated by genes involved in essential and housekeeping processes.^56^ Pangenome studies highlight the potential of this approach to act as a genetic atlas, revealing evolutionary patterns and functional adaptations across species within a given genus.

Advances in genetic tools have made it possible to experimentally validate the function of many genes identified in transcriptomics or comparative genomics studies, connecting hypotheses with the phenotype. The main findings from the last 5 years are listed in Table 1.

**Table 1:** Main Virulence Factors of *P. digitatum*, Mechanisms of Resistance to Fungicides and Host Defense Mechanisms in Citrus, as Revealed by Genomic Approaches and Gene Analyses, from the Past Five Years

Deleted gene (s)	Edition technique	Fuction in virulence/resistance	Reference
*PdCDIE1* (cell death-inducing effector)	Homologous recombination	Significant reduction in virulence; decreased host cell death induction	^46^
*PdGpaA* (Gα protein)	Homologous recombination	Decreased growth, altered cell wall organization, reduced virulence	^70^
*PdMesA* (cell polarity protein)	T-DNA insertional mutagenesis	Defects in polar growth and cell wall integrity; reduced virulence	^40^
*PdXEG1* (endo-β-1,4-glucanase specific for xyloglucan)	Homologous recombination	Impaired growth, development, and virulence	^71^
*mfs2* (MFS transporter)	Homologous recombination	Reduced resistance to prochloraz; altered expression of metabolic genes	^59^
*PdMut3* (Zn(II)2Cys6 transcription factor)	Homologous recombination	Increased virulence; morphological and gene expression alterations	^65^
*pksP* (polyketide synthase)	CRISPR/Cas9	Pigmentation changes; protocol optimization for genome editing	^57^
*arp2* (actin-related protein)	CRISPR/Cas9	Morphological changes; protocol optimization for genome editing	^57^
*PdMFS6* (MFS transporter)	Homologous recombination (ATMT)	25–35% reduction in virulence at early infection stages	^61^
*afpB* (antifungal protein B	Homologous recombination (ATMT)	Contributes to the homeostasis of the cell	^72^
*PdStuA* (APSES transcription factor)	Homologous recombination	Abnormal conidiation, reduced growth, and decreased virulence on citrus fruit	^73^
*Pd1002, Pd1527, Pd1528, Pd1529, Pd1530, Pd1536, Pd1537* (FQ biosynthetic gene cluster)	Homologous recombination (ATMT)	Reduced TQA A biosynthesis; delayed infection onset; smaller lesion size	^55^
*PdatfA* (bZIP transcription factor)	Homologous recombination (ATMT)	Affects growth, pigmentation, and stress response	^66^
*VmaH* (V-ATPase subunit H)	Homologous recombination (ATMT)	Reduced growth, altered intracellular pH, impaired metabolism and decreased virulence	^63^
*Pdgart* (GAR-transferase)	Homologous recombination (ATMT)	Impaired growth, metabolism, mitochondrial function and reduced virulence	^64^

Studies employing suppression subtractive hybridization (SSH) (differential gene isolation) and transcriptional profiling identified *P. digitatum* genes that are upregulated during the initial stages of infection, which is a critical period for successful pathogenesis. These genes are involved in transcription and regulation, and in producing plant cell wall-degrading enzymes, such as pectin methyl esterase. This enzyme is highly expressed in more virulent strains and is induced during infection.^23^

Some research has focused on membrane transporters, such as those in the Major Facilitator Superfamily (MFS). Several MFS transporters in *P. digitatum* have been identified and characterized, revealing the diversity of functions within this transporter family. For instance, studies on four MFS transporters, PdMFS2, PdMFS3, PdMFS4, and PdMFS5, demonstrated that, while not all contribute to fungicide resistance, deleting each transporter individually impacted the fungus’ ability to infect citrus.^47^ Similarly, a transcriptomic analysis of a fungal strain with a defective PdMFS2 gene revealed that this transporter may regulate the expression of multiple drug efflux pump and metabolic genes, contributing to resistance to prochloraz. Thus, MFS transporters are a diverse group of proteins that contribute to fungal virulence and resistance to chemical compounds in various ways.^59^

Further insights into this family have been provided by the characterization of PdMFS6, a recently identified transporter associated with both fungicide resistance and virulence.^61^ Deletion of PdMFS6 reduces pathogenicity in citrus fruits, whereas its overexpression enhances the severity and persistence of the disease under chemical stress. Notably, PdMFS6 is transcriptionally activated during host infection, supporting its role in fungal adaptation to the host environment.^61^

In addition to MFS transporters, other membrane-associated systems have also been implicated in fungal virulence. In particular, vacuolar H ± ATPases (V-ATPases) are essential for intracellular pH homeostasis and cellular function.^62^ In *P. digitatum*, silencing of the V-ATPase subunit H (VmaH) results in reduced growth and pathogenicity, accompanied by alterations in vacuolar pH and fungal morphology. At the molecular level, disruption of V-ATPase activity affects nutrient metabolism and membrane transport processes, impairs mitochondrial function, and increases reactive oxygen species accumulation. These changes are associated with reduced activity of cell wall-degrading enzymes, ultimately compromising the ability of the pathogen to colonize host tissues.^63^

Additionally, enzymes involved in central metabolism also contribute to fungal virulence. In *P. digitatum*, glycinamide ribonucleotide (GAR) transferase, a key enzyme in the de novo purine biosynthesis pathway, plays a critical role in fungal development and pathogenicity. Deletion of the corresponding gene (*Pdgart*) results in severe defects in hyphal growth, conidiation, and germination, which are associated with impaired purine synthesis and reduced ATP production.^64^ Furthermore, disruption of this pathway affects mitochondrial function and energy metabolism, leading to decreased organic acid production and reduced activity of cell-wall-degrading enzymes, ultimately attenuating virulence during citrus infection. These findings highlight the importance of primary metabolic pathways as key determinants of fungal pathogenicity.^64^

Transcription factors also represent key regulators of the pathogen-fruit interaction, such as the *PdMut3* gene.^65^ This gene encodes a putative Zn­(II)­2Cys6 transcription factor, which is a unique family of regulators present in fungi. Surprisingly, eliminating the *PdMut3* gene increased virulence during citrus infection, particularly in the initial phases (three to 4 days after inoculation). This finding contrasts with the role of other transcription factors that promote virulence and is counterintuitive. Deleting the *PdMut3* gene caused a 35% reduction in growth in minimal medium but not in PDA medium and resulted in severe morphological alterations in hyphae and conidiophore development.^65^

Other regulatory families have also been explored in *P. digitatum*. Notably, members of the bZIP transcription factor family have been identified at the genomic level and are known to participate in key processes such as development, stress adaptation, and pathogenicity. A genome-wide analysis revealed multiple bZIP genes in *P. digitatum*, and functional characterization of the conserved transcription factor PdatfA demonstrated its involvement in vegetative growth, pigmentation, and oxidative stress responses.^66^

Analyzing the host’s molecular responses provides valuable insights into *P. digitatum*’s infection strategy and the defense mechanisms activated by citrus fruits. In this interaction, the fungus employs a wide array of virulence factors and regulatory proteins to suppress host defenses and promote colonization. Conversely, citrus fruits activate transcription factors such as CsWRKY65 and CsWRKY25 to strengthen defense signaling and restrict fungal invasion.^67,68^

CsWRKY65 expression is significantly and positively regulated in the peel of citrus fruit infected with *P. digitatum*, increasing drastically within 2.5 dpi. CsWRKY65 binds directly to the promoters of CsRbohB, CsRbohD, CsCDPK33, and CsPR10, activating their transcription. Transient overexpression of CsWRKY65 in citrus peels improves disease resistance by promoting ROS accumulation and pathogenesis-related protein synthesis. Concurrently, CsWRKY65 activates the enzymatic activities of superoxide dismutase (SOD), catalase (CAT), PAL, and POD. These enzymes help maintain ROS homeostasis and prevent oxidative damage.^68^

Similarly, the expression of CsWRKY25 is positively regulated in infected citrus peel. CsWRKY65 and CsWRKY25 both act as transcriptional activators in the nucleus. CsWRKY25 activates the expression of CsRbohB, CsRbohD, and CsPR10. The transient overexpression of CsWRKY25 in citrus plants increased resistance to *P. digitatum* and caused the accumulation of H_2_O_2_ and lignin. Accumulation of ROS activates the antioxidant system, resulting in the positive regulation of genes and the enzymatic activities of cinnamyl alcohol dehydrogenase (CAD), CAT, PAL, and POD. Additionally, the positive regulation of the MPK5 and MPK6 genes suggests that CsWRKY25’s regulatory role may be related to phosphorylation.^67^

Another transcription factor that plays a pivotal role in citrus defense is CsAP2L, recently identified as a key regulator of resistance to green mold.^43^ CsAP2L belongs to the AP2/ERF family and contains two conserved AP2 DNA-binding domains. The protein localizes to the nucleus of citrus peel cells, where its expression increases markedly around 2–2.5 dpi with *P. digitatum*, indicating an active defense response triggered by pathogen invasion. Functional assays using transient overexpression of *CsAP2L* demonstrated enhanced fruit resistance to green mold, evidenced by a reduced incidence and smaller lesion diameters. Molecular analyses further revealed that CsAP2L enhances resistance by modulating the transcription of genes involved in lignin biosynthesis and reactive oxygen species (ROS) metabolism. Mechanistically, CsAP2L binds directly to the promoters of its target genes through GCC-box and AT-rich elements, thereby activating transcriptional cascades that fortify citrus peel tissues against fungal infection.^43^ Importantly, the activity of these defense-related transcription factors must be tightly regulated to maintain immune homeostasis. In this context, Chen et al. identified the E3 ubiquitin ligase CsRGLG4 as a critical regulator of CsAP2L activity.^69^ CsRGLG4 interacts directly with CsAP2L and targets it for degradation via the ubiquitin–proteasome system (UPS), thereby suppressing the transcription of CsAP2L-regulated genes.^69^ This dynamic regulatory mechanism highlights how citrus fruits balance defense activation, preventing excessive responses that could be detrimental to host tissues.

Genomic and gene analysis studies deepen our knowledge of *P. digitatum* biology and its interaction with the host. These studies also reveal potential targets for developing new, effective control strategies. Such targets include virulence factors involved in the pathogen-host interaction. These factors include genes that regulate transcription and the production of enzymes that degrade the plant cell wall, such as pectin methyl esterase.^23^ Other targets include membrane transporters from the major facilitator superfamily (MFS), such as PdMFS2, PdMFS3, PdMFS4, PdMFS5, and especially PdMFS6. These transporters contribute to fungal virulence and resistance to chemical compounds.^47,59,61^ Transcription factors are also targets. One such factor is the recently identified *PdMut3*. Surprisingly, elimination of this transcription factor can increase virulence during citrus infection.^65^ It must be mentioned that despite the increasing number of *P. digitatum* genes that have been functionally characterized, none of them alone is determinant of the virulence of the fungus, a fact that points to the multilayered nature of virulence in this necrotrophic fruit pathogen.

Collectively, genomic and functional gene analyses have substantially deepened our understanding of the molecular basis underlying *P. digitatum* virulence and its interaction with citrus fruit. The integration of high-quality genome assemblies, gene-editing technologies, and multiomic approaches has enabled the identification of key genetic determinants associated with pathogenicity, stress adaptation, and fungicide resistance. In practical terms, these advances provide a robust foundation for the development of next-generation disease control strategies, including the design of targeted antifungal compounds, the disruption of critical virulence pathways, and the enhancement of host resistance through molecular breeding or biotechnology-based approaches. Ultimately, continued exploration of genomic resources and functional gene networks will be essential to improving the long-term effectiveness of postharvest disease management in the citrus industry.

## 4 Biological Control Strategies: Combatting Green Mold in Citrus Fruits

The use of biocontrol microbial agents, such as bacteria, yeasts, and fungi, has become an alternative technique for managing diseases in agriculture, as these microorganisms are generally considered safer alternatives, in addition to reducing the potentially harmful effects of chemical fungicides.^74,75^ However, biocontrol practices are still used on a smaller scale compared with chemical methods, mainly due to their variable efficacy under different climatic conditions.^76^

Among the main mechanisms of action of biocontrol agents (BCAs) are competition for space and nutrients, hyperparasitism, antibiosis, the production of secondary metabolites, competition for iron, lipopeptide production, and the induction of disease resistance in the host.^77–79^ These mechanisms are widely described for BCAs in combating pathogens that cause postharvest deterioration.^80^ In addition, more recent studies have elucidated the involvement of biofilm formation, quorum sensing, oxidative bursts, and the production of antifungal volatile organic compounds (VOCs) in pathogen suppression.^81–83^ Biofilm formation enhances microbial adhesion and persistence on fruit surfaces, creating a stable and competitive niche that prevents pathogen colonization and infection. Quorum sensing allows microbial populations to communicate through signaling molecules (autoinducers), regulating the coordinated expression of genes related to antifungal metabolite and lytic enzyme production, thereby amplifying antagonistic activity. Oxidative bursts, often induced by beneficial microorganisms in host tissues, result in the accumulation of reactive oxygen species (ROS), which damage fungal membranes and cell walls while triggering host-defense responses.^81^ The emission of antifungal VOCs directly inhibits fungal spore germination, disrupts hyphal growth, and interferes with cellular redox homeostasis.^83^ These processes significantly contribute to the suppression of fungi, strengthening the action of biocontrol agents and promoting an unfavorable environment for the development of pathogens.^82^

Iron competition has emerged as a particularly relevant antagonistic mechanism in postharvest systems, especially for yeast-based BCAs, such as *Metschnikowia pulcherrima*. Recent evidence demonstrates that *M. pulcherrima* exhibits superior iron sequestration capacity compared with major citrus pathogens, including *P. digitatum*, *P. italicum*, and *Geotrichum citri-aurantii*. The addition of exogenous Fe^3+^ markedly reduces its biocontrol efficacy, highlighting iron deprivation as a key determinant of the antagonism. This yeast produces pulcherrimin, an iron-chelating pigment, and is able to reutilize pulcherrimin-bound iron through specific transport systems, whereas the pathogens show limited or no ability to access this iron pool. Notably, *P. digitatum* is particularly sensitive to iron limitation, reinforcing the ecological relevance of iron competition during citrus fruit colonization.^79^

Lipopeptide production represents another key antagonistic mechanism employed by bacterial BCAs, particularly members of the genus *Bacillus*. Recent studies have demonstrated that lipopeptides produced by marine *Bacillus amyloliquefaciens* HY2–1 exhibit strong antifungal activity against *P. digitatum* both in vitro and in vivo.^78^ These amphiphilic compounds primarily target fungal cell membranes, leading to increased membrane permeability, lipid peroxidation, reduced ergosterol biosynthesis, and leakage of intracellular nucleic acids and proteins. In addition to direct membrane disruption, HY2–1 lipopeptides induce oxidative stress in *P. digitatum* by triggering intracellular ROS accumulation and suppressing antioxidant defenses, thereby disturbing cellular redox homeostasis. Importantly, in vivo assays on citrus fruits demonstrated that HY2–1 lipopeptides effectively suppressed green mold development and simultaneously activated host defense responses, as evidenced by increased levels of total phenols and flavonoids in citrus peels. This dual mode of action, direct antifungal activity combined with the induction of fruit defense responses, highlights lipopeptide production as a highly effective and multifunctional mechanism in postharvest biocontrol strategies.^78^

Biocontrol agents (BCAs) can act through multiple complementary mechanisms, combining the previously described strategies with the production of antimicrobial compounds. These metabolites are capable of inhibiting or suppressing pathogen growth even at low concentrations, thereby enhancing the overall efficacy of biocontrol.^84,85^ Among them, bacterial isolates often play a particularly prominent role, as they not only form biofilms and communicate via quorum sensing but also secrete lytic enzymes, such as chitinases and nucleases, which degrade key structural components of fungal pathogens. Effective strains are typically strong colonizers, exhibiting rapid proliferation, metabolic versatility, and consistent antimicrobial activity, underscoring the importance of integrating in vitro screening with in vivo validation for the accurate selection of potent biocontrol agents (BCAs).^76^

Potential biocontrol agents against *P. digitatum* are extensively documented in the scientific literature, highlighting various species of microorganisms for their effectiveness in combating this pathogen (Table 2).

**Table 2:** Biocontrol Agents (BCAs) Used Against *P. digitatum*

	Microbial antagonists	Inhibition in vivo	Mechanisms	Source of isolate	Reference
Bacteria	*Bacillus sp*.	89.3%	Secondary metabolites	Soil	^86^
	*Bacillus subtilis*	-	Secondary metabolites	Citrus plants	^87^
		-	Secondary metabolites and lytic enzyme	Saline environments	^88^
	*Bacillus altitudins*	76.8%	Secondary metabolites	Not specified	^89^
	*Pseudomonas isolates*	85.0%	Volatile compounds, siderophores and chitinolytic activity	Soil	^90^
	*Streptomyces nonsenses*	71.5%	Secondary metabolites and enzymatic activity	Soil	^91^
Yeasts	*Candida oleophila*	95.0%	Lytic enzyme and biofilm	Citrus fruits	^92^
	*Clavispora lusitaniae*	87.5%	Biofilm	Citrus packinghouse	^93^
Fungi	*Trichoderma harzianum*	80.0%	Chitinase and glucanase activity	Soil	^94^
	*Nodulisporium sp*.	>70%	Volatile compounds	Medicinal plant	^95^

Lu et al., demonstrated the efficacy of the bacterium *Bacillus altitudins* h217 as a promising biocontrol agent against *P. digitatum*.^89^ In vitro experiments revealed that *B. altitudins* h217 significantly inhibited the growth of *P. digitatum*, forming a pronounced inhibition zone, which underscores its strong antifungal activity. This strain produced highly effective volatile organic compounds (VOCs) in controlling the pathogen, inhibiting *P. digitatum* growth by 76.83%. Important antifungal compounds were identified by gas chromatography-mass spectrometry (GC-MS), including 5-methyl-2-hexanone, methoxyphenyl oxime, and 2-pentylfuran. Additionally, it was found that *B. altitudins* h217 destabilizes the integrity of the cell membrane of *P. digitatum*, induces the production of ROS, and reduces the pathogen’s POD activity, weakening its defense mechanisms and resulting in its inhibition.^89^

Another study demonstrated that a bacterium isolated from the soil of the citrus rhizosphere, phylogenetically identified as *Streptomyces monashensis* h114, exhibited significant antifungal activity against *P. digitatum*. Genomic analysis of the strain revealed the presence of 35 groups of biosynthetic gene clusters for secondary metabolites, responsible for antibiotic biosynthesis. Culture filtrates (CFS) fermented for 36 h showed strong antifungal activity, even after exposure to boiling water and high concentrations of trypsin. Additionally, treatment with CFS caused degradation of the pathogen’s mitochondria and reduced the expression of key developmental genes, such as *PdBrlA* and *PdWetA*. In in vivo experiments, it was observed that CFS increased the activities of defense enzymes, such as SOD and POD, resulting in a significant reduction in pathogen spread and disease incidence.^91^

The mechanism of action of the yeast *Clavispora lusitaniae* AgL21 as a biocontrol agent against *P. digitatum* in lemons was evaluated on the basis of various inhibition strategies. The yeast demonstrated its effectiveness in biofilm formation, wound colonization on lemons, and inhibition of spore germination of the pathogen. Additionally, the efficacy of combining *C. lusitaniae* AgL21 with killer yeast strains of species *Kazachstania exigua* was analyzed. In vivo assays revealed that the combination of these strains enhanced inhibitory effects, achieving 100% efficiency in suppressing green mold compared to 87.5% efficiency when *C. lusitaniae* AgL21 was applied alone. The combined application of the yeasts demonstrated a synergistic effect, resulting from the integration of their multiple mechanisms of action. This approach maximizes the potential of biocontrol by leveraging the complementary characteristics between the strains for a more efficient suppression.^93^

Although yeasts and bacteria have received more attention as biocontrol agents against *P. digitatum* in postharvest citrus fruits, some studies highlight the use of fungal strains. Among these studies, the fungus *Purpureocillium lilacinum* has emerged as a promising biocontrol agent against *P. digitatum*. The filtrate of *P. lilacinum* demonstrated significant antifungal activity, completely inhibiting the growth and spore germination of *P. digitatum* at a concentration of 64%. This inhibition primarily occurs due to the destruction of the fungal cell membrane, resulting in increased membrane permeability and elevated levels of malondialdehyde (MDA), an indicator of cellular damage.^96^ Furthermore, the filtrate significantly reduced the ergosterol content in *P. digitatum*, an essential component of the fungal cell membrane, with an inhibition rate of 81.1% at a concentration of 32%. This reduction resulted in a substantial decrease in the incidence and severity of green mold on the treated oranges compared to the controls. The treatment also increased the activity of key defense-related enzymes in the fruit, such as PAL, PPO, and POD, in addition to promoting greater expression of the corresponding genes, thereby strengthening the plant’s resistance to infection.^96^

The use of microorganisms or their metabolites in agriculture represents a more sustainable alternative to chemical fungicides, which should be progressively adopted to ensure a continuous yield of crops. Significant advances have been made in understanding BCAs for combating the green mold. However, the limited availability of accessible and effective BCAs, together with their inconsistent performance under commercial conditions, still hinders the widespread implementation of biological control.^97^

To overcome these limitations, recent research has shifted toward microbiome-informed strategies that better reflect the ecological complexity of the natural systems. In this context, it is important to consider the microecology of citrus postharvest environments, where the fruit surface (carposphere) hosts a diverse and dynamic microbiota composed of bacteria, yeasts, and filamentous fungi.^98^

Synthetic microbial communities (SynComs) have emerged as a promising approach to address these challenges. SynComs are rationally designed consortia that aim to mimic key functional traits of natural microbiomes by combining microorganisms with complementary mechanisms, including nutrient competition, antibiosis, and induction of host resistance.^99^ Advances in microbiome-based approaches have enabled the selection of core microbial members based on abundance, co-occurrence patterns, and functional potential, providing a more systematic framework for community design.^75^

Although the application of SynComs in postharvest systems is still in its early stages and remains relatively underexplored, emerging studies have provided compelling evidence of their potential. For instance, Jing et al. demonstrated that a synthetic bacterial community derived from citrus epiphytic microbiota significantly enhanced the suppression of postharvest pathogens, including *P. digitatum*, exhibiting greater efficacy and stability compared with individual strains.^100^ Notably, the consortium maintained disease control over extended storage periods, highlighting the importance of synergistic interactions among its members in sustaining biocontrol activity.^100^ These findings reinforce the potential of SynComs as a robust, microbiome-informed strategy for improving the consistency and reliability of biological control in citrus.

Biological control represents a promising and sustainable strategy for managing green mold in citrus fruits, particularly in light of the limitations associated with the intensive use of chemical fungicides. The application of biocontrol agents, capable of acting through multiple mechanisms, contributes to reducing postharvest disease incidence and improving the fruit quality and shelf life. In practical terms, the incorporation of these approaches into production systems can reduce the development of fungicide resistance, minimize environmental impact, and meet the growing demand for safer and more sustainable agricultural practices. Thus, the advancement and consolidation of biological control are essential to strengthening the efficiency and competitiveness of the citrus industry.

## 5 Future Perspectives

Future progress in *P. digitatum* research will depend on advancing beyond predominantly descriptive studies toward integrative frameworks that more effectively connect molecular pathogenicity, host defense regulation, ecological interactions, and sustainable disease management. Although substantial advances have clarified key virulence factors and host resistance mechanisms, important gaps remain regarding the coordinated regulation of fungal secondary metabolism, the dynamic interplay of phytohormones during infection, and the ecological functions of citrus-associated microbiota in disease suppression under postharvest conditions.

Key future research hotspots include deciphering how virulence determinants, stress adaptation pathways, and host responses function as interconnected systems across infection stages and storage environments. In parallel, expanding the exploration of native citrus microbiota and rationally designing microbiome-informed biological control strategies, including synthetic microbial communities (SynComs), may offer more stable, multifunctional, and sustainable alternatives to conventional fungicide-dependent management. Bridging these mechanistic discoveries with ecological validation, formulation technologies, and scalable commercial implementation will be essential to transform next-generation biological control into practical postharvest solutions. Collectively, integrating molecular, ecological, and translational perspectives is expected to shape more resilient and environmentally responsible strategies for citrus green mold management while supporting long-term citrus quality and global food security.

## Supplementary Material



## Notes

Overview of *Penicillium digitatum* pathogenicity, host interactions, molecular tools, and biocontrol strategies relevant to citrus green mold disease­(PDF)
